# First experience with a new negative pressure incision management system on surgical incisions after cardiac surgery in high risk patients

**DOI:** 10.1186/1749-8090-6-160

**Published:** 2011-12-06

**Authors:** Andrea Colli

**Affiliations:** 1 Department of Cardiac Surgery, Hospital Universitari Germans Trias i Pujol, Badalona, Spain; 2 Cardiac Surgery Unit, Department of Cardiology, Thoracic and Vascular Sciences, University of Padua, Padua, Italy

**Keywords:** incision, wound healing, negative pressure wound therapy, cardiac surgery, median sternotomy

## Abstract

**Background:**

Sternal wound infection remains a serious potential complication after cardiac surgery. A recent development for preventing wound complications after surgery is the adjunctive treatment of closed incisions with negative pressure wound therapy. Suggested mechanisms of preventive action are improving the local blood flow, removing fluids and components in these fluids, helping keep the incision edges together, protecting the wound from external contamination and promoting incision healing. This work reports on our initial evaluation and clinical experience with the Prevena™Incision Management System, a recently introduced new negative pressure wound therapy system specifically developed for treating closed surgical incisions and helping prevent potential complications. We evaluated the new treatment on sternal surgical incisions in patients with multiple co-morbidities and consequently a high risk for wound complications.

**Methods:**

The Prevena™incision management system was used in 10 patients with a mean Fowler risk score of 15.1 [Range 8-30]. The negative pressure dressing was applied immediately after surgery and left in place for 5 days with a continuous application of -125 mmHg negative pressure. Wounds and surrounding skin were inspected immediately after removal of the Prevena™ incision management system and at day 30 after surgery.

**Results:**

Wounds and surrounding skin showed complete wound healing with the absence of skin lesions due to the negative pressure after removal of the Prevena™ dressing. No device-related complications were observed. No wound complications occurred in this high risk group of patients until at least 30 days after surgery.

**Conclusions:**

The Prevena™system appears to be safe, easy to use and may help achieve uncomplicated wound healing in patients at risk of developing wound complications after cardiothoracic surgery.

## Background

The incidence of sternal wound infection (SWI) after cardiac surgery ranges from 1-10% depending on the definitions applied and the subset of the population analyzed [1,2]. Despite the use of prophylactic systemic antibiotics, postoperative sternal wound infection still occurs in some circumstances and it continues to be a serious problem after surgical cardiac procedures. Sternal wound infection is associated with significant reduction in quality of life, additional expenses increased length of stay in the hospital and in an increased mortality during the first year [1,3-5]. It has been estimated that SWI may almost triple the costs for patients undergoing coronary artery bypass graft [4-8]; additional costs of up to 80.000 USD have been reported [9].

It has been shown that there is an increased risk of infection in patients presenting with diabetes, smoking, obesity, harvesting of bilateral internal mammary arteries, increasing number of grafts, peripheral vascular disease, renal failure, chronic pulmonary disease, increased duration of mechanical ventilation and preoperative malnutrition [3,10-13]. High-risk patients can be identified using for example the Fowler score permitting the application of additional precautions to help prevent this type of complications [13].

Various studies have focused on techniques or devices to reduce SWI such as microbial sealant prior to surgery [14], different methods of sternal closure (Robicsek technique [15], rigid-plate sternal fixation [16], nitinol clips [17]), different methods of skin closure (intradermic closure [18], skin staples [19], liquid skin adhesive [20], prophylactic gentamycin-collagen sponge [21], topical application of autologous blood products [22], hydrocolloids [23], growth factors [24]) or a combination of them. All these techniques have showed good results reducing incidence of mediastinitis and reducing the length of stay in the hospital. Unfortunately, no one has achieved widespread use due to the absence of strong evidence based on well performed controlled clinical studies and due to the increased costs.

Based on the worldwide positive experience with negative pressure wound therapy (NPWT) for the management of soft tissue defects and the proposed mechanisms of action several authors have used the beneficial effects of NPWT for the management of postoperative incision wounds [25-31].

As a result of the increased use of NPWT for (prophylactic) treatment of closed incisions the manufacturer of the vacuum assisted closure system (VAC). Therapy system recently developed a negative pressure system specifically for treatment of closed incisions. In this study we present our initial experience with the new Prevena™Incision Management System (Kinetic Concepts Inc, San Antonio, TX) in patients, which are at high risk for developing surgical wound complications after cardiothoracic surgery. Our objective was to test the ease of use and safety of the new device in this indication. Secondary endpoints were the occurrence of complications to the wound and the intact surrounding skin.

## Methods

This prospective cohort study was conducted between January 2011 and March 2011 at the Hospital Universitari Germans Trias i Pujol in Badalona, Spain. A total of 97 cardiothoracic procedures were performed during this 3 months period. Ten patients who presented an increased estimated risk for postoperative SWI based on the risk score described by Fowler [13] were consecutively preoperatively elected for treatment with the Prevena™ incision management system. Patients were selected if reached a minimum preoperative Fowler score of 7 (estimated probability of infection of 2.1%). The median estimated risk for SWI was 12.5 points, with a mean of 15.1 and a standard deviation of 6.8. More background patient characteristic can be found in table 1. Based on the Fowler estimated probability of infection score the average risk of infection for these 10 patients was 6.4% (range 2.3% - 16.2%) [13].

**Table 1:** Patient characteristics

PATIENT	AGE, GENDER	PREOPERATIVE VARIABLES	OPERATION	FOWLER SCORE	PROBABILITY OF INFECTION
1	73 y, male	Diabetes, Chronic lung disease, Myocardial infarction	CABGx3 (LIMA-LAD)	11	3.5
2	67 y, male	Diabetes, Chronic lung disease, BMI 30 to 40 kg/m2, Peripheral vascular disease	CABGx1 LIMA-LAD AVR	18	8.5
3	63 y, male	Chronic lung disease, Peripheral vascular disease, Myocardial infarction	CABGx3 (LIMA and RITA)	8	2.3
4	78 y, female	Diabetes, Renal failure, BMI 30 to 40 kg/m2, Peripheral vascular disease	CABGx1 (LIMA-LAD) and AVR	23	12.5
5	65 y, female	Diabetes, Renal failure, BMI 30 to 40 kg/m2, Peripheral vascular disease, Myocardial infarction, Cardiogenic shock	CABGx1 (LIMA-LAD) and MVR	30	16.2
6	62 y, male	Diabetes, Peripheral vascular disease, Myocardial infarction	CABGx3 (LIMA and RITA)	9	2.7
7	72 y, female	Diabetes, Peripheral vascular disease, Myocardial infarction	CABGx3 (LIMA-LAD)	13	4.5
8	62 y, female	Diabetes, BMI 30 to 40 kg/m2, Peripheral vascular disease, Myocardial infarction	CABGx3 (LIMA and RITA)	15	6.0
9	65 y, female	Diabetes, Peripheral vascular disease, Myocardial infarction	CABGx3 (LIMA and RITA)	12	4.0
10	55 y, male	Diabetes, BMI 30 to 40 kg/m2, Peripheral vascular disease, Myocardial infarction	CABGx3 (LIMA and RITA)	12	4.0

y, year; BMI, body mass index; CABG, coronary artery bypass graft; AVR, aortic valve replacement; MVR, mitral valve replacement; LIMA, left internal mammary artery; RITA, right internal mammary artery; LAD, left anterior descending coronary artery.

**Figure 1:**
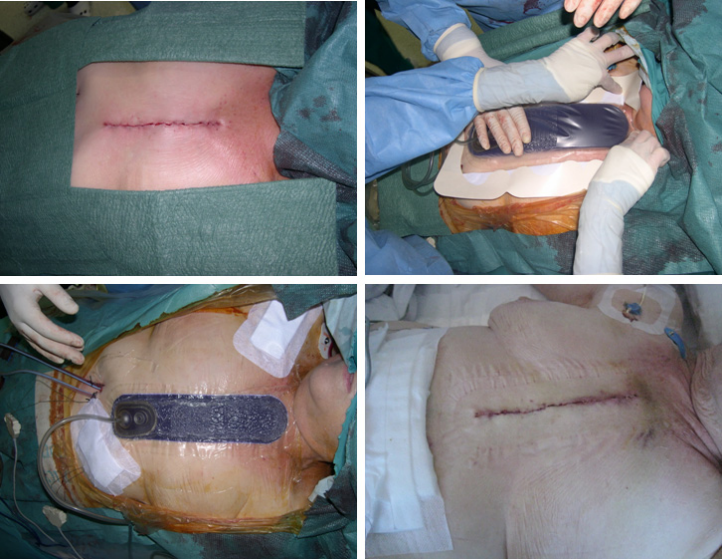
**The clinical application of the Prevena™ Incision Management System**.

Immediately after the surgical procedure all incisions were closed in a standard fashion with 6 to 9 stainless steel wires, one-layer double suture technique with a 1/0 monofilament absorbable suture (Ethibond^®^, Ethicon, Norderstedt, Germany) for the muscular and subcutaneous tissues and one-layer single intradermic suture with 3/0 absorbable suture for the dermis (Monocryl^®^, Ethicon, Norderstedt, Germany). The Prevena™ incisional dressing system was applied in the operating theatre immediately after wound closure. The 24 cm long Prevena™ dressing was placed over the closed incision and negative pressure was applied (Figure 1). The Prevena™ dressing system was always used for 5 days. Patients were monitored daily for symptoms of wound infection. Wounds were inspected immediately after removal of the Prevena™ therapy system and at day 30 after surgery.

This Prevena™ Incision Management system consists of a suction unit with a small canister for fluid collection, a tubing and a sponge dressing with non adherent layer and semipermeable adhesive drape. The dressing contains ionic silver (0.019%) to prevent bacterial growth in the dressing. The system is supplied with a carrying case to minimize effects on patient mobility. The system when applied and activated maintains a closed environment and removes fluids via the application of negative pressure therapy.

According to the manufacturer in order to get a maximum benefit, the Prevena™™ Incision Management System should be applied immediately after the surgery to closed surgical incisions. The incision site must be cleaned and dried to ensure proper fixation of the transparent adhesive drape, chest drainage tubes and pain management control devices should be located away from dressing application site to prevent air leakage via these materials. The Prevena™ dressing tube is connected to the Prevena™ suction unit by the canister for fluid collection. The vacuum therapy unit produces a negative pressure of -125 mmHg and its lifespan is 8 days. Light showering is permitted. It must be continuously active for a period between 3 and 5 days and with a maximum of 8 days. The Prevena™ Incision Management System should not be used to treat open or dehiscent surgical wounds or in patients with a incision that produces an excessive amount of exudate which may exceed the capacity of the Prevena™™ canister (maximum capacity 45 mL). Other NPWT Systems should be considered for treating these wounds. It's use in patients with sensitivity to silver is contraindicated, and it should be avoided in case of ischemia in the incision area, inadequate hemostasis of the incision and cellulitis of the incision area. Written informed consent was obtained from the patient for publication of this case report and accompanying images. The present clinical research was carried out in compliance with the Helsinki Declaration. A copy of the written consent is available for review by the Editor-in-Chief of this journal.

## Results and Discussion

Application of the Prevena™ dressing system immediately after wound closure was uncomplicated and took only a few minutes. In two cases, after starting the negative pressure therapy, the audio and visual alarms were activated due to an air leak. In both cases the incision dressing was partially applied over the chest drainage tube and a small bleeding in that area produced the loss of the vacuum in the dressing. We removed the excessive dressing and we resealed the Prevena™ system with patch strips. In both cases this resolved the problem and the vacuum therapy continued to work properly until its removal at day 5. In the other 8 cases no leakage or other alarms were reported.

Upon removal of the dressing after 5 days of continuous Prevena™ therapy the wound healing was complete in all patients. No wounds developed complications such as infection. Re-evaluation of the wounds at day 30 again showed complete wound healing with absence of wound infections.

Wound repair is an orchestra of biological and molecular events such as cell migration, proliferation and of extracellular matrix depositioning and remodeling. Certain pathophysiologic and metabolic conditions can alter this normal course of events so that healing is impaired or delayed. This may result in acute or chronic non healing wounds.

The risk of developing a surgical wound infection is largely determined by 3 factors: 1) the amount and type of microbial contamination of the wound, 2) the condition of the wound at the end of the surgical procedure, and 3) host susceptibility, which is the patient's intrinsic ability to deal with microbial contamination. These factors interact in a complex manner. Measures intended to prevent surgical wound infections are directed to all 3 factors. Since most infections are acquired in the operating room and in the intensive care unit, measures for prevention should be addressed to influence the practices of the entire surgical team.

Measures aimed at preventing microbial contamination of the wound are the following: preparation of the patients before operations (treating active infections, maintaining a short preoperative stay in the hospital, antiseptic skin preparation), preparation of the surgical team (scrubbing hands with sterile materials), preparation and maintenance of the operating room environment (reducing airborne microorganisms), operative technique (surgery duration, wound drainage, wound closure, strict glucose control), wound care (wound dressing), prophylactic antimicrobials, protection of patients from other infected patients or personnel, surveillance and classification.

Intravenous antibiotic prophylaxis in cardiac surgery achieves up to a five-fold reduction in surgical wound infections rate [32]. However, the use of broad-spectrum antibiotics for surgical prophylaxis adds to the selective pressure driving the development of resistant pathogens [32]. It has been reported that patients with antibiotic resistance bacteria have a higher mortality, prolonged hospitalization and increased health-care costs compared with those infected with non-resistant pathogens [33].

Various studies have focused on techniques for reducing SWI after cardiac surgery such as microbial sealant prior to surgery, different methods of sternal closures, different methods of skin closures, and a combination of them.

However, no one has achieved widespread use due to the absence of strong evidence based on well performed controlled clinical studies.

Two articles were published recently showing some evidence on the proposed mechanisms of action of the Prevena™ dressing system. Wilkens et al [29] using a finite element model showed that the negative pressure applied with this system decreased lateral tissue stresses and changed the direction of tissue stresses to a distribution that is typical for intact tissues. Bench testing results indicated that the dressing bolsters appositional forces at the incision. Kilpadi et al [30] showed in a porcine study that using the Prevena™ dressing system hematoma and seroma formation was reduced and that the negative pressure dressing increased lymph clearance. In a recently published RCT Pachowsky et al [31] studied the incidence and volume of seromas when the Prevena™ incision management system was applied to surgical wounds after total hip arthroplasty procedures and compared these with the use of standard wound treatment after THA. Similarly to the porcine study also this RCT in humans showed a significant reduction in seromas and in seroma volume upon application of the Prevena™ incision management system.

In four recent studies a modified topical Vacuum Assisted Closure system was used and a non-adherent layer was applied between the foam and the incision and surrounding intact skin.

Stannard et al [25] published results of a randomized controlled trial (RCT) demonstrating decreased drainage and improved wound healing following negative pressure wound therapy treatment of both hematomas and severe fractures. In another RCT Stannard et al [26] compared the effect of NPWT at -125 mmHg to standard postoperative dressings used in closed incisions following high-energy trauma. Two-hundred-sixty-two patients were included in the study, of those 141 were randomized to NPWT and 121 were randomized to control treatment. The incidence of wound dehiscences and infections was found to be lower in the NPWT group compared to control group (p < 0.03 and p < 0.02, respectively). Atkins et al [27] reported results of a retrospective review using NPWT at -125 mmHg for 4 days on 57 patients at high risk for sternal wound infections after cardiac surgery. These patients were compared to 213 patients without infection risk of and managed with standard of care procedures. The authors observed no complications in the NPWT group and one infection in the control group, concluding that the NPWT was easily applied and well tolerated and should be considered for patients with an increased risk for sternal wound infections. Gomoll et al [28] reported a case series of 35 patients of high-risk patients following orthopedics surgery. NPWT was applied at -75 mmHg for 3 days. No infections were observed after a minimum follow-up of 3 months.

The Prevena™ Incision Management System represents the first dressing system specifically designed to help prevent the development of healing complications such as infection and dehiscence. In particular the use of Prevena™ intends to improve the incisional healing with an innovative and effective negative pressure dressing via protection from external contamination, keeping the wound edges together, equalizing strain in the tissue and via application of the beneficial effects of the NPWT therapy (stimulation of cell proliferation, reduction of inflammatory mediators, increasing arterial and subcutaneous oxygen partial pressure, reduction of the wound stress) already demonstrated over the past 15 years of clinical experience with this technology.

This report presents our initial application and evaluation of the Prevena™ Incision Management System in our institution and represents the first report in cardiac surgery patients in Europe on this new class II device. The results observed using the Prevena™ system are encouraging. In this very high risk for SWI group of patients there were no early or late wound infections. We are aware that the small number of patients is a limitation of the present evaluation. Due to the fact that we did not perform a large controlled clinical trial we are not able to identify the Prevena™ system as the only factor that could explain the observed favorable results.

We estimate that the Prevena™ Incision Management System may play an important role in patients presenting a preoperative or intraoperative high risk of SWI (double mammary harvesting, obesity, severe chronic obstructive pulmonary disease, malnutrition, long hospital stay), together with other clinical strategies such as weight loss and/or smoking, interventions targeting Staphylococcus aureus, including nasal decolonization and vaccines. Due to the high costs of sternal wound infections and the increased risk in this population additional expenditures to help prevent complications might be justified. More robust controlled clinical and economical studies are required to assess the clinical and cost-effectiveness of the Prevena™ Incision Management System and to determine which patients would benefit most from this new specialized wound technology.

## Conclusion

In conclusion the preliminary results observed in our center are encouraging; however, our data are insufficient to at this moment recommend a widespread use of this technology. Our data can serve as a basis for more definitive studies to determine which patients and wound types could benefit from this therapy.

## Abbreviations

AVR: aortic valve replacement; BMI: body mass index; CABG: coronary artery bypass graft; cm: centimeter; LAD: left anterior descending coronary artery; LIMA: left internal mammary artery; mmHg: millimeters mercury; MVR: mitral valve replacement; NPWT: negative pressure wound therapy; RCT: randomized clinical trial; RITA: right internal mammary artery; SWI: sternal wound infections; THA: total hip arthroplasty; USD: United States dollar; y: year; RCT: randomized controlled trial.

## Competing interests

The author declares that he has no competing interests.
