# Correction: first experience with a new negative pressure incision management system on surgical incisions after cardiac surgery in high risk patients

**DOI:** 10.1186/1749-8090-7-37

**Published:** 2012-07-13

**Authors:** Andrea Colli, Maria-Luisa Camara

**Affiliations:** 1 Department of Cardiac Surgery, Hospital Universitari Germans Trias i Pujol, Badalona, Spain; 2 Cardiac Surgery Unit, Department of Cardiology, Thoracic and Vascular Sciences, University of Padua, Padua, Italy

## Correction

The first publication of the work [1] did not present one of the authors’ name. Maria-Luisa Camara has now been correctly added to the author list, and the Competing interests and Authors’ contributions sections have been updated to reflect this.

## Competing interests

Maria-Luisa Camara does not have any competing interests to be declared.

## Authors’ contributions

AC: wrote the manuscript and was the major contributor to the conception, design and data acquisition for this study. MLC: operated on the majority of patients and contributed to the conception, design and data acquisition (pictures) of this study. All authors read and approved the final manuscript.

## References

[B1] Colli A First experience with a new negative pressure incision management system on surgical incisions after cardiac surgery in high risk patients J Cardiothorac Surg 2011 6 160 10.1186/1749-8090-6-160 22145641 PMC3305521

